# Wireless Low-Power Integrated Basal-Body-Temperature Detection Systems Using Teeth Antennas in the MedRadio Band

**DOI:** 10.3390/s151129467

**Published:** 2015-11-20

**Authors:** Chin-Lung Yang, Gou-Tsun Zheng

**Affiliations:** Department of Electrical Engineering, National Cheng-Kung University, Tainan 70101, Taiwan; E-Mail: n26000321@mail.ncku.edu.tw

**Keywords:** implantable fractal dental antenna, basal body temperature, temperature compensated voltage-controlled oscillator, thermal sensor

## Abstract

This study proposes using wireless low power thermal sensors for basal-body-temperature detection using frequency modulated telemetry devices. A long-term monitoring sensor requires low-power circuits including a sampling circuit and oscillator. Moreover, temperature compensated technologies are necessary because the modulated frequency might have additional frequency deviations caused by the varying temperature. The temperature compensated oscillator is composed of a ring oscillator and a controlled-steering current source with temperature compensation, so the output frequency of the oscillator does not drift with temperature variations. The chip is fabricated in a standard Taiwan Semiconductor Manufacturing Company (TSMC) 0.18-μm complementary metal oxide semiconductor (CMOS) process, and the chip area is 0.9 mm^2^. The power consumption of the sampling amplifier is 128 µW. The power consumption of the voltage controlled oscillator (VCO) core is less than 40 µW, and the output is −3.04 dBm with a buffer stage. The output voltage of the bandgap reference circuit is 1 V. For temperature measurements, the maximum error is 0.18 °C with a standard deviation of ±0.061 °C, which is superior to the required specification of 0.1 °C.

## 1. Introduction

Modern medical, electronic, and wireless communication technologies have developed rapidly in the last two decades. Implantable medical devices for monitoring health have attracted considerable attention for remote home care applications. Numerous implantable devices such as retinal prostheses, glaucoma monitoring, cochlear implants, cardiac pacemakers, implantable cardiac defibrillators, and blood coagulation detection devices [1,2,3] have been proposed for diverse medical applications.

General implantable biomedical devices measure the voltage or current by using biosensors related to those physiological signs of interest. The voltage or current is sampled with the circuit and controls the oscillator to process the frequency modulation (FM) followed by a microwave power amplifier and then radiated through an antenna for transmission. Both amplitude shift keying and frequency shift keying are popular ways of transmitting modulated signals, however, due to the complex and varying biological activities and environments, frequency modulated signals exhibit considerable uncertainty resulting from the attenuation of intensity, interferences, or noise. Therefore, frequency modulation is suitable for use in biomedical telemetry devices.

The implantable chip used in this study can measure physiological basal body temperature (BBT) signals, thus enabling monitoring of the female endocrine systems [4,5]. The BBT can help women determine their health status in terms of pregnancy, miscarriages, progesterone deficiency, and other physiological conditions, so users can understand their physical conditions to achieve early detection, prevention, and treatment of diseases, however, the BBT scale is not convenient for long term daily measurements.

In this study, the telemetry system is composed of an implantable antenna and low-power, low-voltage circuits including a sampling amplifier, voltage control oscillator, and bandgap reference. The bandgap voltage circuit produces a stable reference voltage to supply the power of the sensor system. A ring oscillator is used to achieve a small size, low power consumption, and easy implementation. A bandgap voltage reference is applied to generate and supply a stable and temperature-compensated current source to the oscillator.

Implantable biomedical devices must take into account the variations of the physiological environment. The complementary metallic oxide semiconductor (CMOS) parameters and circuit characteristics vary with the temperature of the biological environment or the heat produced during the chip operation. The temperature could severely affect all circuits, especially the oscillator. This would reduce both the thermal measurement accuracy and the FM-based communication system. Several temperature-compensated circuits are proposed to solve the problem of the frequency drift caused by the temperature [6,7], so the output frequency of the oscillator is not affected by temperature variations. In this study, not only are a thermal sensor sampling circuit and the transmitter introduced, but also a temperature compensation circuit to solve the problem of frequency drifts caused by the temperature.

The article describes a wireless temperature sensor with a temperature-compensated voltage-controlled oscillator (VCO). The design of the temperature-sensing system is described in Section 2, including a sampling circuit and a low-power voltage-controlled oscillator with temperature compensation. The experimental results and discussion are provided in Section 3. Finally, Section 4 provides the conclusions, indicating that the proposed design is a viable approach for low-power wireless BBT-sensing systems.

## 2. System Architecture

**Figure 1:**
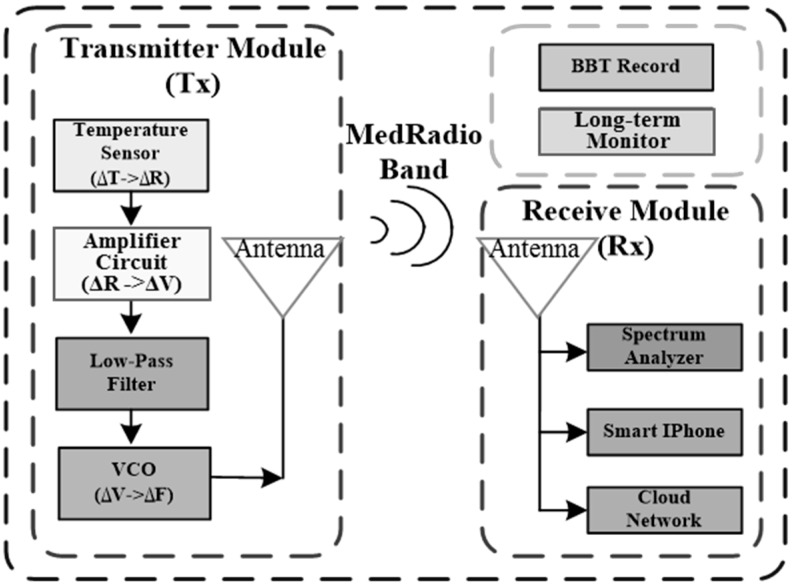
Block diagram of the wireless BBT measurement system.

The proposed wireless platform of the BBT temperature-sensing systems is shown in Figure 1. Thermistors as temperature sensors detect the thermal variation, and the resistance change corresponds to the voltage change (ΔV), which modulates the voltage-controlled oscillator that operates in the MedRadio band (401–406 MHz) (ΔF), resulting in a simple FM transmitter. The radio waves can be further propagated using a miniature MedRadio band antenna [8] to complete a wireless low-power compact transmitter. The implantable antenna is 8 × 11.5 × 8 mm^3^. The area of the antenna is only 245 mm^2^ because of the fractal design. The measured antenna gain is −6.78 dBi. On the receiver end, a receiving antenna is attached on a spectrum analyzer to record the central frequency and demodulate the sampling temperature. To improve the signal quality, signal processing may be applied after receiving the measured signal. For example, a simple moving algorithm can store the slow time-varying information and suppress high frequency noise.

### 2.1. Implantable Antennas

The first limitation when designing an implantable device is the size of the antenna. Based on the half wavelength of the MedRadio band (401–406 MHz), approximately 35 cm, the common size is too large to be a feasibly implantable size. Besides, shrinking the antenna aperture also reduces the antenna efficiency and bandwidth. In this context, the first dental antenna proposed by Yang can be sufficiently compact to be implantable in the oral cavity [8]. The choice of size is based on the consideration of the size of the average molars of Asians which is approximately 8 × 11.5 × 8 mm^3^. All-ceramic dentures are one of the most suitable synthetic materials, and zirconium dioxide (ZrO_2_, ε_r_ = 21) is chosen due to that ZrO_2_ also has a high dielectric substrate which facilitates a feasibly compact antenna and also provides optimal biocompatibility.

**Figure 2:**
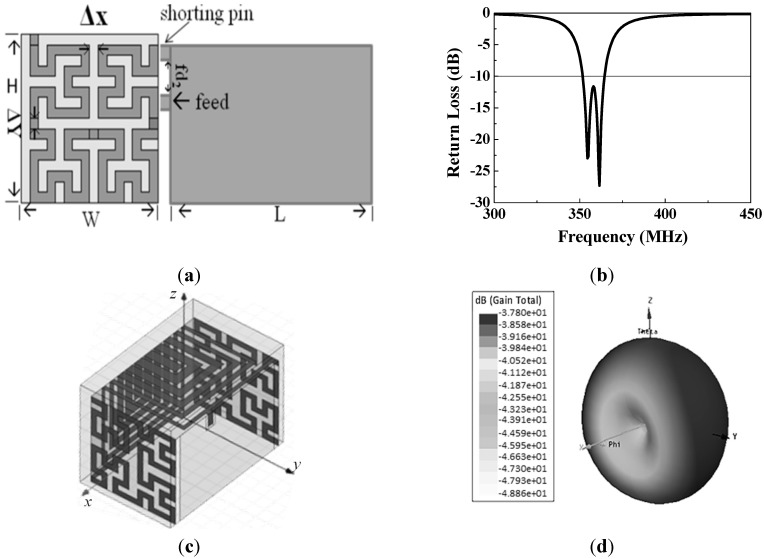
Proposed dental fractal antennas in BBT system (**a**) Hilbert fractal structure; (**b**) Simulation result of the antenna return loss; (**c**) Whole miniature 3D dental antenna structure; (**d**) radiation pattern.

To enable the antenna to operate in the MedRadio band (401–406 MHz), the principles used to miniaturize dental antennas are important. Based on the high dielectric constant substrate, the Hilbert fractal and the spiral architecture, and three-dimensional antenna folding technologies, the implantable antenna is successfully miniaturized within the constrained size of 8 × 11.5 × 8 mm^3^. The Hilbert-based fractal antenna is proved to achieve a significantly lower resonant frequency. Figure 2a shows a standard planar inverted-F antenna (PIFA) antenna whose radiator part is modified according to the Hilbert fractal structure which is part of the basic elements of the antenna. The right side of Figure 2a is a ground that can be faced to the tongue side. The spiral architecture coupled the other resonance mode to expand the bandwidth [8]. A full-wave simulator ANSYS HFSS high frequency structure simulator (HFSS) was performed for the design to optimize the proper dimensions and to fine tune the operational frequency. In the simulation results shown in Figure 2b, the bandwidth was coupled by two resonant modes and increased to 13.8 MHz. Therefore, a broadband output is fulfilled successfully in the miniaturized antenna by combining spiral and Hilbert curves in the design. The center frequency is further tuned after considering the biological environment and the implementation concerns. Figure 2c illustrates the complete 3D structure of the miniature dental antenna. The radiation pattern is shown in Figure 2d. The antenna gain is measured to be −6.78 dBi, which is also much better than that of traditional implantable antennas (~−20 dBi) at 400 MHz [8].

### 2.2. Thermal Sensor Readout Circuit

**Figure 3:**
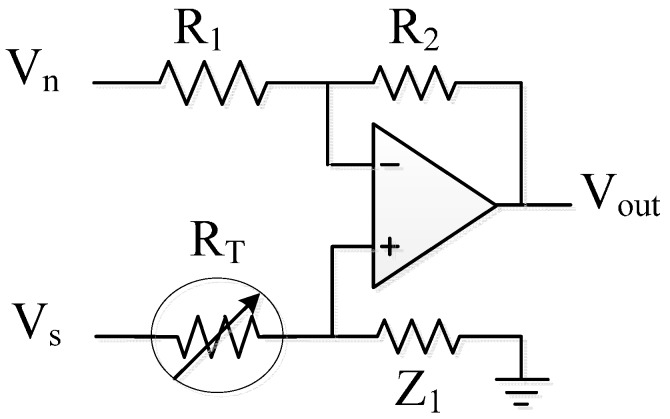
Active bridge circuit for the thermal sensor readout circuit.

To construct thermal sensors suitable for embedding in general devices, the physiological sensor must be small. A surface-mount device thermistor (PTS0805, Vishay, Shelton, CT, USA) is chosen. Its volume is 2 × 1.25 × 0.45 mm^3^. The temperature detection is designed to be a high-accuracy measurement of the human body temperature, which typically ranges from 36 to 37.5 °C so the chosen resistor has corresponding values from 570 to 573 Ω over these temperatures. There is only a small variation of the resistor of approximately 3 Ω (0.5%). Typical resistance conversion circuits for reading the thermistor are the Wheatstone bridge, resistive subdivision, constant current methods, active bridge, and negative impedance converter [3]. Negative impedance conversion provides high sensitivity, but the operation conditions should be carefully adjusted to prevent saturation. For the popular and practical Wheatstone circuit, a high-accuracy galvanometer should be applied. Because of the small variation, an operational amplifier (Op-Amp) is employed for a boosted voltage signal. The active bridge circuit is shown in Figure 3. V_+_ of the active bridge circuit (Figure 3) is not directly grounded; the noise can be reduced compared to the inverting configuration Op-Amp with a fixed reference current. Although such a simple active bridge circuit is not the most sensitive sampling circuit, the active bridge is suitable for stable control to enlarge the sampled resistance variation. A proper operation range is adjusted by V_n_. In this design, precision resistance alloys are chosen for implementation due to high accuracy, stability, and a low temperature coefficient. The active bridge circuit in this paper is designed to have a gain of 100, so 3-Ω thermistor variation can corresponds to the 0.2 V to 0.8 V at the output of the readout circuit for the input of the VCO.

To enhance the response to the resistance change, an active bridge circuit is commonly used due to the noise immunity and low power consumption. V_+_ of the Op-Amp is not directly grounded and can reduce the interference or noise from the ground. The voltage gain is determined using the ratio R_2_/R_1_, and the sensitivity is varied by Z_1_. However, the operating range of the input V_s_ may be limited due to the saturation of the high-sensitivity Op-Amp, and V_n_ can be included to adjust the output voltage range.

### 2.3. Proposed VCO with Temperature Compensation

**Figure 4:**
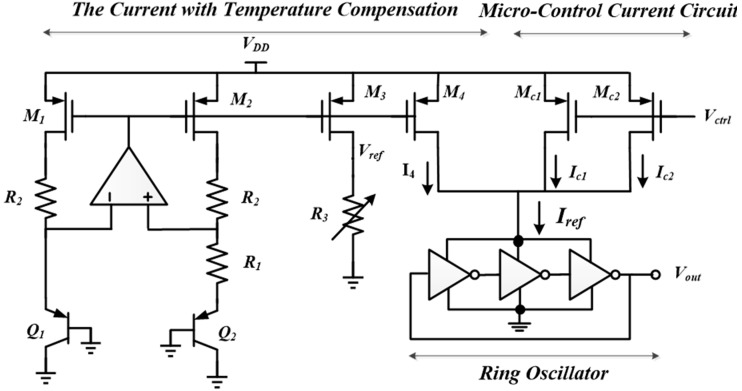
VCO with a temperature compensation circuit.

For thermal sensor measurement, it is crucial to design a temperature-compensated transmitter to prevent the output frequency from being affected by the temperature of the transmitter. The bias current is the major affected factor when the performance of an oscillator circuit is varied with the temperature. This study uses bandgap reference circuit characteristics to implement a temperature-independent current source. The consistent output current source is fed to the ring oscillator as a tail current *I*_ref_ to stabilize the oscillation frequency. The circuit schematic is shown in Figure 4. One useful solution is to enforce the bias current coming from a stable bandgap reference of which the principle combines a positive and a negative temperature coefficient [9]. However, the output voltage (V_BE_ + *K*V_T_) produced by the traditional bandgap reference voltage is approximately 1.25 V, and it is not suitable for the low-power circuits. The voltage divider method can achieve a low voltage portion, but the quiescent current leakage wastes valuable harvested power and reduces the efficiency. In [10,11,12], temperature compensated ring oscillators are studied. In [10], the principle of a 1-V voltage reference can be achieved using a nearly zero temperature coefficient current source, followed by an I/V converter with a resistor. The proposed thermal-sensing system uses low-voltage bandgap reference circuit characteristics to implement the temperature-independent current source. The consistent output current source is fed to the ring oscillator as a tail current *I*_ref_ to stabilize the oscillation frequency. Based on the bandgap reference voltage circuit, the current *I_4_* is mirrored with M_3_, M_4_, and R_3_, according to Equation (1):

(1) $$V_{ref}=V_{BE1}+(V_{T}\cdot \ln n)\frac{R_{2}}{R_{1}},\;I_{R3}=I_{ref}=I_{4}=\frac{V_{ref}}{R_{3}}$$

where *n* is the ratio of *I*_s1_/*I*_s2_, and the constant of *R*_2_/*R*_1_ (ln *n*) is tuned to approximately 17.24 to achieve a zero temperature coefficient.

A ring oscillator functions as a frequency modulation circuit and is biased using the temperature-compensated current source. The delay time of the inverter is modified by the current amount through the inverter *M*_c2_ to control the frequency of the ring oscillator in a 400-MHz band. The VCO architecture can be divided into two parts: (a) coarse tuning systems, which use the resistor R_3_ to adjust the output current I_4_. The current controls the conversion time of the whole ring loop to reach the center frequency of 400 MHz. (b) Fine-tuning systems: M_c1_ and M_c2_ of the two p-type metal-oxide-semiconductors (PMOSs) are used to adjust the microcurrent variation and to modulate the output frequency range to within 401–406 MHz. PMOS is used due to its low noise, and the PMOS substrate body is an N-type well, and the noise immunity is superior compared to p-type metal-oxide-semiconductors (NMOS). The control voltage *V*_ctrl_ bridles the M_c1_ and M_c2_ to produce an output frequency range of 401–406 MHz. Hence, the small current changes can precisely control the VCO to cover the required bandwidth. The simulated power consumption is 661 µW, and the output power is −3 dBm.

The ring oscillator is used to transmit the measured temperature from the thermal sensors; hence, a temperature-independent current source must be involved. The output reference voltage variation is less than 1 mV, the simulated temperature variation coefficient is 2 ppm/°C, and the voltage variation coefficient is 0.42%/V. After temperature compensation, from a temperature range of 0 to 50 °C, the drift current is less than 0.01 μA and the current variation coefficient is 195.3 ppm/°C. The constant voltage source is set to 0.5 V to control the output frequency, and the corresponding frequency variation coefficient is 10.1 ppm/°C.

## 3. Results and Discussion

### 3.1. Circuit Blocks Measurement Results

**Figure 5:**
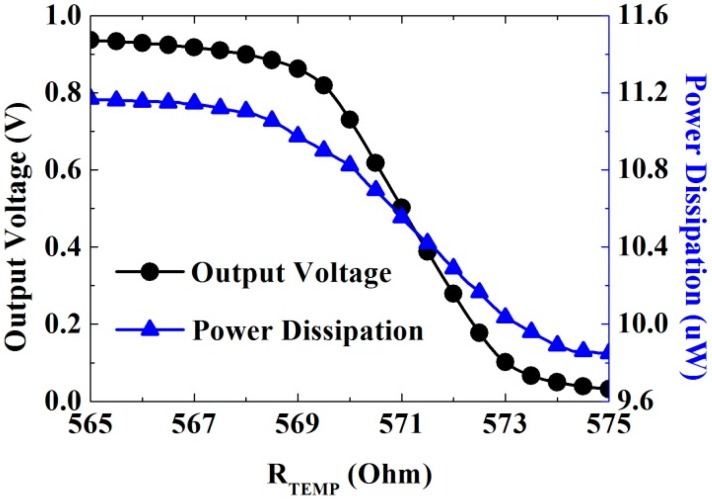
Measurement of the output voltage with *R*_temp_.

The thermal readout circuit is implemented and measured on a FR4 PCB (Kinsten International, Shenzhen, China) test board. The resistance of the PTS0805 thermistor (Vishay, Shelton, CT, USA) ranges from 570 to 573 Ω, which corresponds to 36–37.5 °C. The output voltage of the readout circuit is from 0.03 to 0.92 V, as shown in Figure 5, and the maximal power consumption is only 11.2 μW.

### 3.2. Measurement Results of the VCO

**Figure 6:**
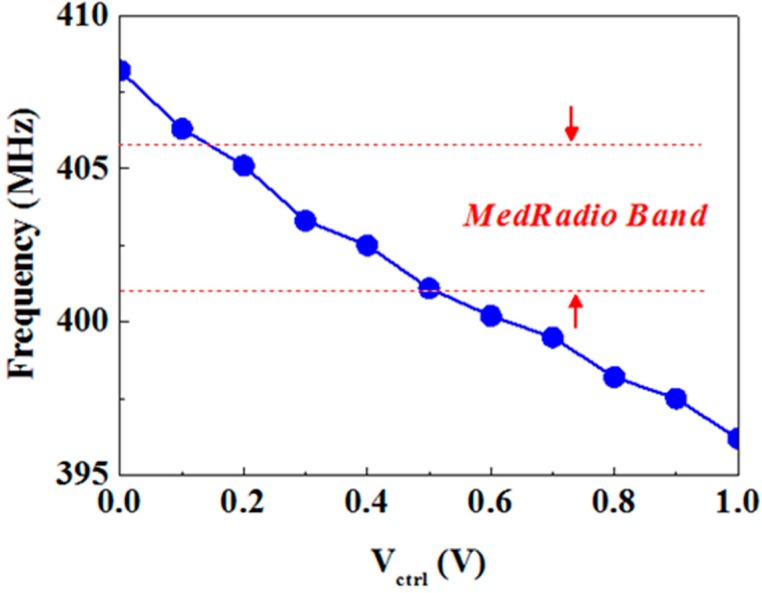
Measured output frequency operating in the 400-MHz band.

The test chip of the VCO was fabricated using Taiwan Semiconductor Manufacturing Company (TSMC) 0.18-μm standard CMOS technology. Figure 6 presents the measured output frequency of the VCO. When the different control voltage ranges from 0.2 to 0.5 V, the frequency of the VCO output corresponds at 401–406 MHz. The power consumption is measured to 4.014 mW including the output buffer which can be implanted using an inverter. The output power is measured −3.04 dBm at *V*c = 0.9 V by using an Agilent (Santa Rosa, CA, USA) N9010A EXA Spectrum Analyzer (9 kHz–26 GHz). The performance of the VCO is also compared with that of other implantable VCOs. The proposed VCO shows temperature invariance and remains in low-power operation.

### 3.3. Wireless Measurement of the BBT Range

**Figure 7:**
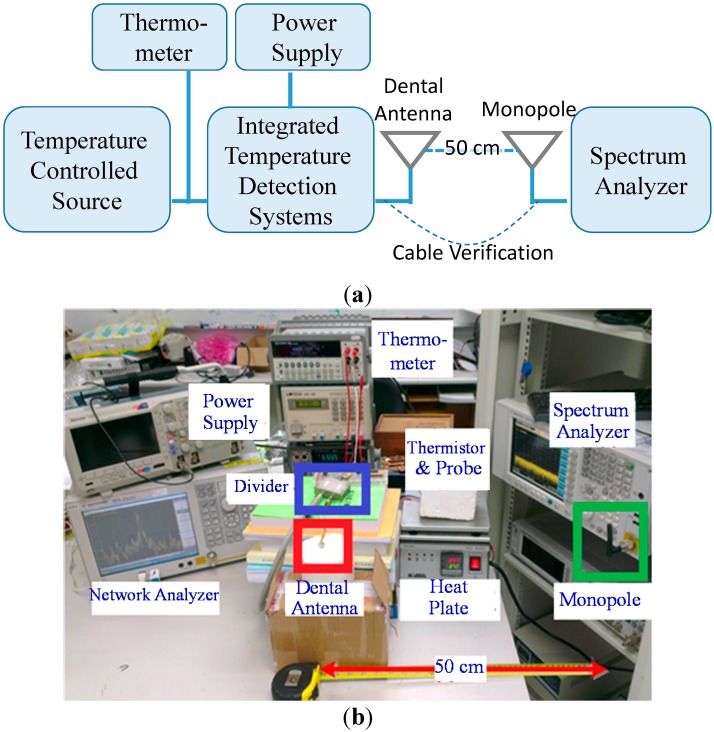
Measurement setup of the temperature-monitoring system. (**a**) Overall measurement diagram; (**b**) Measurement setup picture.

Figure 7 shows the overall measurement setup for temperature verification. First, the thermistor is placed on the heating plate for temperature control, and a calibrated thermometer that detects temperature by using an M3300A digital multimeter (thermometer, Keithley, Cleveland, OH, USA) is attached for accurate temperature reference. Multiple samples were performed to validate this proposed integrated system. The calibration is based on a baseline experiment to extract the least square error coefficients of the linear relationship which is correlated the measured frequency with the setting temperature. Each measurement requires multiple samples that last longer than 5 s to ensure a stable temperature. A calibration table of the temperature is then established and correlated with the frequency. On the basis of the measured frequency, the temperature can be estimated.

**Figure 8:**
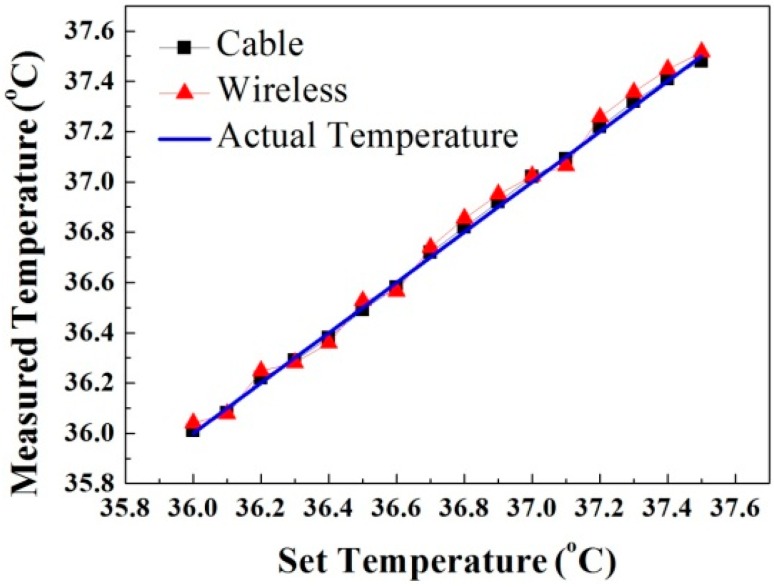
Comparison of the measured and actual temperatures.

The final measurement result is shown in Figure 8. The wireless telemetry measurement at a distance of 50 cm is compared to the wired-transmission (cable) data and the actual temperature. The errors mainly come from the varying wireless environment and some random system noise. The maximum error of the temperature measurement is 0.18 °C at 36 °C. The maximum error range is ±0.5%, and the average error is ±0.061 °C. The system satisfies the accuracy requirement of 0.1 °C.

## 4. Conclusions

This paper presents a wireless 1 V low-power temperature-monitoring system operating in a MedRadio band. The biomedical device can be mounted on the bodies of subjects to perform temperature monitoring and transmit data wirelessly. The main requirement of the performance metrics is low power consumption, low voltage, and the low dependence on temperature. The design is introduced and discussed in this paper. The area and power consumption are strictly limited in the implanted device. First, the sampling amplifier circuit is explored and analyzed, and precision resistance is chosen to achieve the high sensitivity of the amplification circuit.

The system power is supplied and conditioned by the bandgap reference, and the output voltage is 1 V. The architecture output voltage is not limited to 1.25 V as a typical bandgap reference and does not vary with temperature variation and the supply voltage. The voltage-controlled oscillator is designed based on the MedRadio band with a low temperature coefficient, comprising a temperature-independent current source and a fine-tuned ring oscillator. The output frequency ranges over the MedRadio band, and the power consumption is < 40 μW/4 mW without/with the buffer, respectively.

Finally, the temperature is measured and transmitted wirelessly to obtain a precise temperature estimate from the thermistors. The maximal error is 0.18 °C, and the system average error is ±0.061 °C. Therefore, the proposed system is suitable for a low-power implantable telemetry device to promote biomedical applications and to facilitate remote home care for early disease prevention and treatment.
